# Diabetic kidney disease; review of the current knowledge

**DOI:** 10.12861/jrip.2013.24

**Published:** 2013-06-01

**Authors:** Heshmatollah Shahbazian, Isa Rezaii

**Affiliations:** ^1^Diabetes Research Center, Ahvaz Jundishapur University of Medical Sciences, Ahvaz, Iran; ^2^Department of Nephrology, Ahvaz Jundishapur University of Medical Sciences, Ahvaz, Iran

**Keywords:** Diabetic kidney disease, Chronic kidney disease, End-stage renal disease

## Abstract

Diabetes is the most common cause of chronic kidney disease (CKD) and end-stage renal disease (ESRD) in most parts of the world. 20 to 30% of diabetic patient have diabetic nephropathy in type 1 and type 2. Hyperglycemia is the key of nephropathy creation. Hyperglycemia also by production of toxic materials, advanced glycosylated end product (AGE), increased activity of aldose reductase has some role. Some metabolites of arachidonic acid, hemodynamic derangements and genetic factors have also some role. Although diabetic nephropathy is most common cause of nephropathy in these patients, but diabetic patients are also prone to other urinary tract and renal parenchymal disease and should not be confused with renal failure due to diabetic nephropathy. The principle of treatment of diabetic nephropathy is based on tight control of hyperglycemia, tight control of blood pressure and glomerular pressure, control of dyslipidemia, restriction of protein intake and smoking withdrawal.

**Implication for health policy/practice/research/medical education:**

Diabetes is the most common cause of chronic kidney disease (CKD) and end-stage renal disease (ESRD) in most parts of the world. 20 to 30% of diabetic patient have diabetic nephropathy in type 1 and type 2. The principle of treatment of diabetic nephropathy is based on tight control of hyperglycemia, tight control of blood pressure and glomerular pressure, control of dyslipidemia, restriction of protein intake and smoking withdrawal.

## Introduction

Kidneys are one of the important organs that are involved in diabetes. Diabetes is the most common cause of end-stage renal disease (ESRD) in most parts of the world. Kidney involvement both directly and indirectly increase involvement of other organs and increase morbidity and mortality in diabetic patients.

Since with untreated diabetic nephropathy, a significant decrease in life expectancy of patients happens; therefore, prevention of this debilitating condition and if there is, early diagnosis and treatment is important.

Due to the increasing industrialization of societies, increased immobility and changes in diet and lifestyle and increasing prevalence of obesity, insulin resistance and type 2 diabetes prevalence is increasing.

Because many of the classic symptoms of diabetes in type 2 diabetes, unlike type 1 diabetes, do not occur, the importance of screening methods for identifying these patients and screening them for kidney involvement is very important.

### Epidemiology

Diabetic nephropathy is now the most common cause of chronic kidney disease (CKD). Both types of diabetes can lead to chronic kidney disease and eventually ESRD. But given the much higher prevalence of type 2 diabetes than type 1, often patients with ESRD have type 2 diabetes.

The overall incidence 20 years after diagnosis is approximately 4 to 17% and after 30 years is about 16%.

According to some studies, the incidence of nephropathy in type 1 diabetes is decreasing (1). The main reason for that is early diagnosis of type 1 diabetes and good control of hyperglycemia.

20 to 30% of diabetic patients have diabetic nephropathy in type 1 and 2.

When type 1 diabetic patients are affected with diabetic nephropathy, it is likely to reach ESRD to more than 75% after 20 years. While the risk for type 2 diabetic patients after onset of clinical nephropathy is approximately 20%. Perhaps one of the reasons is that many patients with type 2 diabetes die from cardiovascular events before they reach ESRD.

Clinical renal involvement is usually happens 15 to 25 years after the onset of type 1 and 2 diabetes. Better relationship between the onset of type 1 diabetes and its diagnosis‏ leads to‏ predict the onset of renal involvement in type 1. While in some type 2 diabetic patients in time of diagnosis of diabetes, kidney damage is present (2). So the screening of patients susceptible to type 2 diabetes is important. Authors of the study indicated that 16.7% of patients initially diagnosed with type 2 diabetes had microalbuminuria and 3.3% of patients had serum creatinine above 1.5 mg/dl and were in renal failure (3).

About 30% of patients with type 2 diabetes who are suffering from kidney disease, indeed have other renal disease but no diabetic nephropathy, which is verified by the absence of retinopathy and albuminuria (4).

The peak incidence of nephropathy is 10 to 20 years after diabetes onset and then it occurs as a progressive decrease. So that if the patients spent more than 30 years of his illness and still be normoalbuminuric, it likely to decrease diabetic nephropathy risk (5).

### Pathogenesis and pathophysiology

Hyperglycemia not only causes but also aggravates glomerular lesions. Glomerular lesions may be prevented and if the lesions are present, slowed or stopped with tight control of blood sugar (6). Indeed hyperglycemia is the key of nephropathy creation.

High glucose concentration causes impairment in cell growth and production of growth factors and the occurrence of genes which will ultimately increase the extracellular matrix. Hyperglycemia may be causing toxic products which may lead to cell injury. For example, TGFβ is one of these toxic products. TGFβ increases production of extracellular matrix in the glomerular mesangium. Also with inhibition of collagenases synthesis causes decreased extracellular matrix removal.

The metabolic effects of prolonged hyperglycemia is advanced glycosylated end product (AGE) production. In hyperglycemia some excess glucose binds with amino acids in blood or tissue proteins and ultimately leads to the production of AGE (7).

AGE binds to collagen and cause microvascular complications and renal complications .Long-term hyperglycemia causes structural changes in some proteins that biological activity of these new compounds are high, like HbA_1_c.

Studies show that proteins containing AGE increase protein kinase C activity and appearing of TGFβ. Protein kinase C provides an important role in cell proliferation and differentiation, and apoptosis. This enzyme increases extracellular matrix and cytokines and production of endothelin. This changes causes thickening of the glomerular basement membrane and obstruction of arteries and increased glomerular permeability.

Another important enzyme in the pathogenesis of diabetic nephropathy is aldose reductase. This enzyme converts glucose to sorbitol. Long-term hyperglycemia increases the enzyme activity and sorbitol production, which increases the extracellular matrix. Accumulation of extracellular matrix in the mesangium and glomerular basement membrane (GBM) increase mesangium volume and thickening of GBM.

Some metabolites of arachidonic acid by activating the receptor TXA2 (thromboxane A2) lead to oxidative stress and glomerular injuries (8).

The earliest histological change is thickening of GBM (9). Approximately after 3 to 5 years hyalinization of the afferent and efferent arterioles is seen (10). Increasing mesangium volume is seen about 3 to 5 years after diabetes onset, which sometimes reaches 15 years (11).

In overt nephropathy, both GBM thickening and increased mesangial matrix often are present. Mesangial matrix expansion leads to glomerular sclerosis. In about half of patients glomerular sclerosis has nodular pattern which is known as Kimmelstiel-Wilson nodules that shows severe glomerular sclerosis. Histological lesions pathogenesis are the same in type 1 and 2 diabetes. The main pathological lesions that lead to clinical albuminuria and nephropathy is increased mesangial matrix.

Mesangial matrix expansion strongly increases the development of systemic hypertension and urinary albumin excretion (12,13). The lesion is strongly associated with reduced glomerular filtration.

Other glomerular lesion is podocyte separation of the GBM and decreased foot process that will lead to worsening of proteinuria (14-17).

Another important factor in the development of glomerular lesions is hemodynamic derangements. Auto-regulation of renal blood flow is impaired in diabetic nephropathy, so abnormal dilation of afferent arteriole leads to increased glomerular filtration and this can lead to glomerular sclerosis in long term.

After developing sclerosis blood flow in remaining intact glomeruli increases and causes rise in glomerular pressure so eventually sclerosis.

Systemic blood pressure can also directly transfer to the glomeruli and causes glomerular sclerosis. In type 1 diabetes with microalbuminuria, systemic blood pressure will begin to increases, although it may still be within normal range. Many patients with type 2 diabetes also have essential hypertension that increases after beginning of renal lesions.

### Genetics

Many patients with long-term hyperglycemia have no nephropathy while others with not very long disease course have clinical nephropathy. This may be due to predisposing factors including genetic.

Risk of diabetic nephropathy increases in type 1 and type 2 diabetic patients with a history of diabetic nephropathy in one of their first degree relatives.

One of the genetic factors is angiotensin type 2 receptor gene on the X chromosome.

Male patients with type 1 diabetes who are homozygous for the gene have lower glomerular filtration than heterozygous groups (18). Another effective genetic factor is polymorphism of angiotensin converting enzyme (ACE) gene. Polymorphism of the Deletion/Insertion ACE gene is related strongly with ACE concentration in plasma and risk of coronary artery disease in diabetic and non-diabetic patients. Plasma ACE levels in subjects with the DD genotype is about twice as group II (18).

In a study on type 2 diabetic patients with proteinuria, 95% of individuals with the DD genotype reach ESRD in 10 years and after starting dialysis, patients with DD genotype had higher mortality(19).

Diabetic patients who have a family history of hypertension or cardiovascular disease are more likely to develop nephropathy. Also the polymorphisms of genes related to insulin resistance plays a role in the development of diabetic nephropathy.

### Screening

There are various methods for screening of diabetic nephropathy in patients. It actually starts to prove microalbuminuria, to prevent its progress toward macroalbuminuria (clinical nephropathy), or even turn it into normoalbuminuria status.

Microalbuminuria defines as urinary excretion of 30-300 mg of albumin in the 24 hours urine collection continuously. Macroalbuminuria of clinical nephropathy is when albumin excretion in 24 hours urinary collection be greater than 300 mg continuously(Table 1).

**Table 1:** Classification of albuminuria in diabetic nephropathy

	Spot urine Alb/Cr (mg/g )	24 urine albuminuria(mg)
normal	<30	<30
microalbuminuria	30-300	30-300
macroalbuminuria	>300	>300

Some evidence indicates that the onset of type 1 diabetes before puberty may play a role in creating microvascular complications including nephropathy. Therefore, clinical judgment about when to start screening on every patient should be considered individually. If hyperglycemia, hypertension and lipid disorders are not well controlled, in type 1 diabetic patients, microalbuminuria may develop in less than five years from the onset of diabetes (20). It is logical that screening begins earlier for these patients and patients who diabetes onset is before puberty (21,22).

There are two conventional methods for microalbuminuria screening:

1) Measuring the Alb/Cr ratio in a random urine sample which the morning sample is better.
2) 24-hour urine collection and measurement of albumin.

It should be noted that conventional strip test is not able to detect microalbuminuria. Therefore, negative urine protein analysis with this method does not reject microalbuminuria.

It should be considered that some drugs such as ACEIs and ARBs and non-dihydropyridine calcium channel blockers (verapamil, diltiazem) decrease urinary albumin excretion and may cause the tape test be negative (false negative). Some conditions like high and uncontrolled blood pressure, fever, heavy physical activity, uncontrolled hyperglycemia and acute infections increase transiently in urinary excretion of albumin (false positive).

Because of the large daily changes in urinary albumin excretion rate, to prove the existence of microalbuminuria, test should be repeated three times at intervals of 3-6 months. It needs to demonstrate persistent microalbuminuria with at least two positive tests.

### Natural course

Classification of clinical stages of diabetic nephropathy is as follows:

### First stage: Hyperfiltration

Since the beginning of diabetes, glomerular filtration and renal blood flow increases and the kidneys enlargement (renomegaly) is also seen. The urine albumin excretion (UAE) rate was still less than 30 mg in 24 hour and blood pressure is also normal. The first pathological result is thickening of the GBM. Whatever hyperfiltration rate is more, developing of nephropathy is higher. If the initial glomerular filtration rate (GFR) is more than 150 ml per minute it will increase the chance of diabetic nephropathy (19).

### Second stage: Microalbuminuria

With the progression of kidney involvement, UAE will also increases. It is also called, hidden or subclinical nephropathy. In this stage the conventional strip test is often negative. At this stage the risk of cardiovascular disease begins to increase. Changes in albuminuria rate in time is associated with different risk of decreased kidney function, so in microalbuminuric patients that the rate of albumin exertion increased, constant or decreased, the risk of decreased GFR is very high, moderate and low respectively (23). In type 1 diabetes the prevalence of other microvascular complications such as retinopathy and neuropathy also increases with the increase in albuminuria. Our study showed that 35.2% of patients with type 2 diabetes have microalbuminuria. Also, hyperlipidemia, age, duration of diabetes are risk factors of microalbuminuria (24).

Diagnosis at this stage is a very good opportunity to prevent progression to clinical nephropathy or even back to the normoalbuminuria situation. But without treatment, the risk of developing clinical nephropathy in type 1 diabetic patients after 10-15 years (more than 75%) and in type 2 after 15-20 years (20-40%) is high.

In a study on 75 adult patients with type 1 diabetes who were normoalbuminuric and had normal blood pressure during a follow-up of more than five years, the UAE and their blood pressure were monitored. 24-hour blood pressure (ambulatory) of patients was controlled (25). In the final of study, 14 patients had microalbuminuria, while 61 patients remained normoalbuminuric. The important point is that the average systolic pressure during sleep in microalbuminuric patients was significantly high but was not in normoalbuminuric group. The relative risk of progression to microalbuminuria was measured as systolic blood pressure at night on systolic blood pressure in day.

Ratios equal to or less than 0.9; the natural drop in blood pressure at night, had negative predictive value of 91% for microalbuminuria. Furthermore, the risk of microalbuminuria in the group with equal or less than 0.9, was seventy percent less than the group with more than 0.9. The conclusion is that in type 1 diabetic patients increased systolic blood pressure at night (sleep), is a predictor of microalbuminuria in the future.

If microalbuminuria is associated with retinopathy, the probability that the origin of microalbuminuria is due to diabetes, increases. Conversely, when there is no retinopathy, other causes of CKD are probable.

However, the predictive value of microalbuminuria in diabetic nephropathy in type 1 diabetes is more than type 2,because many type 2 diabetic patients develop microalbuminuria because of hypertension and nephrosclerosis.

### Third stage: Macroalbuminuria

This stage also called clinical nephropathy that occurs about 10-20 years after onset of diabetes, (about 5-10 years after onset of microalbuminuria). In this stage coronary artery disease and cerebrovascular events also increases obviously compared to the previous stage. About 75% of patients in this stage have high blood pressure. Control of blood pressure in type 2 diabetic patients with previous hypertension, is more difficult.

At this stage the conventional dipstick test is positive for proteinuria and urinary albumin excretion rate in 24h collection is more than 300 mg. After this, GFR decline, about 10-12 ml annually.

With the onset of clinical nephropathy and in the absence of therapeutic interventions, progressive decline in kidney function occur and proteinuria increases that can reaches the nephrotic range. Diabetic retinopathy is very helpful to confirm the diagnosis of diabetic nephropathy. Hypertension and proteinuria, independently leads to reduce GFR and progression to ESRD.

20% of type 2 diabetic patients before the onset of clinical nephropathy and 40% of them with clinical nephropathy have renal vascular disease (26).

Tubulointerstitial fibrosis also begins at this stage, and if the extent and severity is more, renal lesions and prognosis will be worse.

In clinical nephropathy patients develop hypoalbuminemia and edema. Serum albumin level in diabetic patients with nephrotic syndrome who have generalized edema is higher than non-diabetic patients with nephrotic syndrome; because glycated albumin in diabetic patients passes easily through the capillary wall, then water shifts from plasma to interstitial space.

Basically this means that in diabetic patients with nephrotic syndrome the edema will develop with milder hypoalbuminemia than nondiabetic patients with nephrotic syndrome (27).In approximately 30-40% of patients with type 1 diabetes, when proteinuria exceeds the range of 300-500 mg in 24 hours, edema occurs around the eyes and ankles, and then generalized edema (27).

### Fourth stage: End-stage renal disease

Almost 20-30 years after onset of diabetes,approximately 10 years after the onset of clinical nephropathy, the patient reaches this stage. The risk for type 1 is more than type 2 diabetes. But the prevalence of type 2 diabetes is 9 times more than type 1, so most diabetic patients with ESRD have type 2 diabetes.

In these circumstances, the mortality rate in diabetic patients is more than nondiabetic patients. In addition cardiovascular and cerebrovascular disease in these patients is higher. In this stage, the incidence of diabetic foot ulcers is higher, which sometimes reach 25%.

In the UKPDS report, annual chance of progression from diagnosis of diabetes to onset of microalbuminuria, from onset of microalbuminuria to overt nephropathy, and overt nephropathy to increase in serum creatinine or switching to renal replacement therapy, is respectively, 2, 2.8, 2.3%.

### Diagnosis

In order to prove the diagnosis of diabetic nephropathy, the following criteria are used:

1)Enough time: At least 10 years past the onset of diabetes, but this criterion is often not verifiable in type 2.
2)Persistent albuminuria more than 300 mg in 24 hours
3)Diabetic retinopathy at the same time**:** Given that almost all patients with type 1 diabetes with diabetic nephropathy have retinopathy, retinopathy in patients with first and second criteria, prove the diagnosis, and lack of it virtually excluded diagnosis.

Considering that only 50-60% of type 2 diabetic have retinopathy at the time of diagnosis of nephropathy, the presence of diabetic retinopathy, confirms the diagnosis, however, its absence does not exclude the diagnosis. If retinopathy was not present, it must first ensure the absence of other renal diseases.

Our study demonstrated that the prevalence of retinopathy in type 2 diabetic patients in microalbuminuria stage was 67% and in macroalbuminuria stage was 86% and the prevalence of diabetic nephropathy in patients with retinopathy was 72%.Also there is a significant relationship between the severity of nephropathy and retinopathy in the non-proliferative diabetic retinopathy but this relationship was not significant in the proliferative diabetic retinopathy (28).

A fundamental question is whether a diabetic patient with advanced diabetic nephropathy have normal urinary albumin excretion rate? The answer is yes (29).

Diabetic patients are also prone to other urinary tract and renal parenchymal disease and should not be confused with renal failure due to diabetic nephropathy. For example, the possibility of acute renal failure following acute pyelonephritis in these patients is more than general population. One of the problems that involves these patients frequently is bladder detrusor muscle dysfunction (neuropathic bladder), that can lead to obstructive nephropathy. In these patients due to renal artery stenosis at the same time, ischemic renal disease is likely, that can lead to acute and progressive or chronic kidney failure. In diabetic patients, type IV renal tubular acidosis (hyporeninemic hypoaldosteronism) is probable, that exacerbate by renin-angiotensin- aldosterone system inhibitor and non-steroidal anti-inflammatory drugs.

The risk of acute tubular necrosis (ATN) after using contrast agents, is high. Also, these patients because of diabetic neuropathy have gastroparesis and vomiting and are prone to hypovolemia and acute renal failure. Systolic heart failure in these patients is due to multiple risk factors that can lead to prerenal failure or ischemic tubular necrosis.Another major complication of diabetes is the papillary necrosis that can lead to obstructive acute renal failure in bilateral ureteral obstruction or unilateral obstruction in one kidney patients or in patients with underlying CKD.

It should be noted that the majority of patients with diabetic nephropathy have neuropathy at the same time. It is not necessary for diagnosis of nephropathy but its absence diminishes the likelihood of diabetic nephropathy. Some criteria that should be mentioned for diagnosis of non-diabetic nephropathy are in Tables 1 and 2.

**Table 2:** Criteria for diagnosis of nondiabetic nephropathy

Absence of retinopathy in type 1
**Renal disease less than 5 years or after 30 years of diabetes onset**
Puria, RBC or WBC cast
Progressive proteinuria
Progressive renal failure
Acute nephrotic syndrome
Acute renal failure
Macroscopic hematuria

It should be noted that microscopic hematuria without RBC cast is seen frequently in biopsy-proven diabetic nephropathy (30). In a study to examine whether can distinguish between glomerulonephritis and diabetic nephropathy, It was found that acantocyturia equal to 5 or more acanthocyte in every 100 red blood cells in patients with clinical diabetic nephropathy is rare. Thus if acantocyturia is present, renal biopsy is required to rule out other glomerular disease (30).

It should be noted that many patients with CKD due to diabetic nephropathy does not have small kidneys. In other words, small kidneys (relative to the stage of renal failure) prove the diagnosis of chronic renal failure in diabetic nephropathy, but lack of small kidneys, does not exclude the diagnosis.

### Treatment

Treatment of diabetic nephropathy is based on some principle:

1)Tight control of hyperglycemia
2)control of blood and glomerular pressure
3)Control of dyslipidemia
4)Restriction of protein intake
5)Smoking withdrawal.

### Tight control of hyperglycemia

Tight control of glucose can prevent microalbuminuria, conversion of microalbuminuria to macroalbuminuria and may be leads microalbuminuria status to normoalbuminuria.The goal is to achieve a HbA_1_c<7% but some offer <6.5%.

The exact treatment of hyperglycemia with insulin can return glomerular hypertrophy and hyperfiltration partially. However, both are risk factors for diabetic nephropathy.

Treatment with insulin can reach blood glucose to almost normal range, so it reduces the onset or progression of nephropathy (31). Also, the exact treatment of hyperglycemia with insulin keep proteinuria in the same level or even reduce its value, although this beneficial effect may be apparent after two years of strict control of blood glucose. During these two years blood glucose should be maintained near-normal.

### Control of blood and glomerular pressure

Hypertension and proteinuria, both progress nephropathy, and lead to ESRD.

Considering the pro-fibrotic effects of angiotensin II on vascular system, inhibit the effect, reduce capillary pressure and also may prevent glomerular lesions.

In addition to exact hyperglycemia control, by strict control of blood pressure and use of renin- angiotensin-aldosterone system inhibitors, risk of nephropathy progression clearly reduce.

All antihypertensive medications are effective in reducing systemic blood pressure, but the most effective drugs in reducing intra-glomerular pressure, are renin-angiotensin-aldosterone inhibitors such as angiotensin converting enzyme inhibitors (ACEIs) and angiotensin receptor inhibitors (ARBs).

When type I diabetic patients suffer from high blood pressure, they usually have reached to clinical nephropathy. While about 30% of type 2 diabetic patients in time of diagnosis of diabetes and about 70% at beginning of diabetic nephropathy have high blood pressure.

Using combination of ACEIs and ARBs reduce UAE in 35% and ESRD in 28% in diabetes type 2 compared using alone (32).

In the prospective, open and controlled study was done by author, combination of losartan 100 mg daily and enalapril 40 mg daily has beneficial effects of antiproteinuria more than monotherapy with either of these drugs in patients with type 2 diabetes. This antiproteinuric effect is independent from antihypertensive effect and in short term can delays kidney failure. This dual therapy did not have risks and side effects more than single treatment (33).

Luno *et al*. evaluated 45 patients with idiopathic proteinuric nephropathy and showed combination of ACEIs and ARBs with lower doses also has similar role (34).

Animal studies also show that low dose dual inhibition reduce angiotensin II activity more than high dose captopril or losartan alone (35).

Some authors have shown anti-proteinuric effects with higher doses. Since proteinuria has important role in renal disease progression, they suggested drug doses can increase to tolerate rate(36-37).

The reason for better efficacy of combination therapy is various mechanisms of these drugs in inhibition of renin-angiotensin system.

Both drugs have direct effect on vascular tone and blood pressure that can have an important role in the persistence of progression of renal disease (38). Angiotensin II also has significant effects on the intrarenal hemodynamic and increase protein excretion through the capillary network and stimulate cellular growth (39).

ACE inhibition cannot completely inhibit the production of angiotensin II but ARBs can fully inhibit the effect of angiotensin I on AT_1_ and this causes the accumulation of angiotensin II that via AT_2_ receptor causes dilation of blood vessels and decreased cell proliferation (40).

On the other hand, the ACE inhibitors and not ARBs reduce degradation of bradykinin that is a vasodilator.

Despite better effects of combination therapy on proteinuria reduction, new studies showed that the effect of dual therapy compared with single therapy has not any different on the primary outcome such as death from cardiovascular diseases, myocardial infarction, stroke or hospitalization because of heart failure.

Moreover, in a large study, the rate of complications and mortality in the dual therapy was higher (41). According to the results of this study, dual therapy is not recommended.

In diabetic patients, except for pregnant women, target blood pressure in normoalbuminuric and microalbuminuric patients is less than 130/80 mmHg and in patients with clinical nephropathy is less than 125/75 mmHg.

Dihydropyridine calcium channel blockers, such as amlodipine and nifedipine, may be worse proteinuria and may progress nephropathy in diabetic and non-diabetic patients. Conversely, non-dihydropyridine calcium blockers such as verapamil and diltiazem, reduce the risk of overt nephropathy and improve size selectivity in glomeruli in diabetic nephropathy. If blood pressure is not controlled with ACEIs or ARBs, diuretics should be added and, if inadequate, non-dihydropyridine calcium channel blockers, such as diltiazem, should be added as well.

Approximately after 5-7 days of prescription of renin-angiotensin-aldosterone system inhibitors, creatinine levels should be controlled. If patient’s baseline creatinine level increases by more than 30%, medication should be stopped and evaluate patient for the bilateral renal artery stenosis, renal artery stenosis in one kidney or even a narrowing of the unilateral renal artery in diabetic patients with CKD. Also should be aware of risk of hyperkalemia and metabolic acidosis (42).

Anti-proteinuric effects of renin-angiotensin-aldosterone system inhibitors is exacerbated with reducing sodium intake and taking other antihypertensive medications at the same time including diuretics and non-dihydropyridine calcium channel blockers.

Even in patients with normal blood pressure those inhibitors reduce proteinuria with reduce intra-glomerular pressure and prevent progression of microalbuminuria to macroalbuminuria and clinical nephropathy.

In AVOID study, aliskiren, as a direct inhibitor of renin secretion was added to losartan, and its effects on reduce proteinuria was evaluated. The results showed that aliskiren plus losartan compared with losartan alone resulted in more than twenty percent reduction in proteinuria without significant effect on systemic blood pressure (43).

Potential benefits of spironolactone as antagonist of aldosterone receptors was tested in a double-blind study on 59 patients with diabetes 2 that were ever treated with ACEIs or ARBs. Spironolactone 50 mg a day were given in treatment group and another group were given placebo. Urine albumin to creatinine ratio up to 40% and systolic blood pressure 7 mmHg and diastolic blood pressure 3 mmHg were reduce in spironolactone group, whereas these parameters did not change in the placebo group (44).

A meta-analysis found that pentoxifylline reduced proteinuria and could have similar effects as ACE inhibitors (45).

One of the problems of diabetics’ patients is hyporeninemic hypoaldosteronism.

About 75% of patients have asymptomatic hyperkalemia. In other cases, symptoms include muscle weakness or arrhythmias are common.

About 50% of these patients have hyperchloremic metabolic acidosis. This syndrome usually occurs in older patients. As we know, with increasing age renin and aldosterone secretion decreases. Also most patients have mild to moderate renal insufficiency. Drugs that increase serum potassium levels should be used with caution in these patients (46). Since beta blockers and calcium channel blockers inhibit renin secretion, it is recommended that these drugs are not taken in patients with hypoaldostronism hyporeninemic.

Also because vasodilators prostaglandin such as PGI_2_, PGE_2_, stimulate renin secretion, non-steroidal anti-inflammatory drugs should be avoided in these patients (45).

If the serum potassium is less than 6 meq per liter, and the disease is asymptomatic, treatment usually do not require. If treatment require, mineralocorticoids (fludrocortison) or the sodium potassium exchange resins [kayexalate (sodium polystyrene)] can use. Both types of treatment in patients with cardiac decompensation may exacerbate previous failure (46).

### Dyslipidemia

The most common lipid disorder in diabetes is increased triglycerides and reduced HDL. Although LDL levels in these patients is usually normal, but it has high atherogenic properties.

Accompaniment of glucose intolerance or diabetes, high blood pressure, low HDL and high triglycerides, obesity and sometimes high LDL are known as metabolic syndrome, will lead to increased risk of cardiovascular disease.

Due to the presence of hypertension, dyslipidemia and diabetes, there is a risk of nephropathy and its progression.

Treatment of lipid disorders reduce microvascular (nephropathy and retinopathy) and macrovascular (coronary, cerebrovascular, peripheral vascular) complication in diabetic patients.

Therapeutic purposes by the American diabetes association are: The blood triglyceride level of less than 150 mg/dL, HDL over 60 mg/dl and LDL less than 100 mg/dL.

In diabetic patients over 40 years age, that their total cholesterol levels is >135 mg/dl, treatment with statins may be useful to reduce LDL by 30% from the baseline value (regardless of its amount) (47).

In the Physician's Health Study that was conducted during fourteen-year follow-up, dyslipidemia was associated with increased risk of renal failure in men who initially had normal renal function (47).

An interesting study was performed about the effects of LDL apheresis on improvement of kidney function in type 2 diabetic patients with nephrotic proteinuria (due to diabetic nephropathy). The first group included five men and three women with a mean age of 54.6 years and the second group included six men and four women, with mean age of 56.6 years. Both groups had prolonged nephrotic syndrome. LDL apheresis effect on proteinuria and urinary podocyte (podocyturia) was studied. The first group receives apheresis and at the end of the study was found that total cholesterol, LDL, LP (a), urea and serum creatinine were significantly lower and creatinine clearance was significantly increased. Also urinary protein excretion rate and urine podocyte decreased in these patients significantly (48).

### Protein restriction

High protein intake can increase glomerular filtration and sclerosis. Studies have shown that reducing protein intake in diabetic patients can reduce proteinuria and slow the progression of renal failure. Protein restriction can lead to decrease symptoms of uremia and delay need for dialysis.

The possible mechanism of effect of protein restriction is reduced hyperfiltration in healthy nephron.

American diabetes association recommends the maximum daily intake of protein is 0.8 g protein per kg for patients who have increased urinary albumin excretion (macroalbuminuria).

However, it must be noted that severe protein restriction increases morbidity and mortality in ESRD patients.

### Stop smoking

In a study effects of smoking on renal complications in 359 type 1 diabetic patients were evaluated. In those who were smokers, prevalence of UAE was higher (2.8 times) than non-smokers. After stopping of smoking, there was a clear improvement in the UAE. Similar results also obtained in type 2 diabetic patients with nephropathy.

## Conclusion

The principle of treatment of diabetic nephropathy is based on tight control of hyperglycemia, tight control of blood pressure and glomerular pressure, control of dyslipidemia, restriction of protein intake and smoking withdrawal.

## Acknowledgements

The authors wish to thanks the following people, staff of diabetes research center for their valuable help and contribution to this manuscript: Drs. Aleali and Drs. Yazdanpanah. We are also grateful to Dr. Rezaii for his unsparing help.

## Authors’ contributions

All authors equally prepared the manuscript.

## Conflict of interests

The author declared no competing interests.

## Ethical considerations

Ethical issues (including plagiarism, informed consent, misconduct, double publication and redundancy) have been completely observed by the authors.

## Funding/Support

None declared.
